# Development and evaluation of RADA-PDGF2 self-assembling peptide hydrogel for enhanced skin wound healing

**DOI:** 10.3389/fphar.2023.1293647

**Published:** 2023-11-28

**Authors:** M. Deptuła, J. Sawicka, P. Sass, P. Sosnowski, P. Karpowicz, M. Zawrzykraj, A. Wardowska, A. Tymińska, M. Dzierżyńska, Z. Pietralik-Molińska, B. Peplińska, J. Zieliński, K. Kondej, M. Kozak, P. Sachadyn, S. Rodziewicz-Motowidło, M. Pikuła

**Affiliations:** ^1^ Laboratory of Tissue Engineering and Regenerative Medicine, Division of Embryology, Medical University of Gdańsk, Gdańsk, Poland; ^2^ Department of Biomedical Chemistry, Faculty of Chemistry, University of Gdańsk, Gdańsk, Poland; ^3^ Laboratory for Regenerative Biotechnology, Faculty of Chemistry, Gdańsk University of Technology, Gdańsk, Poland; ^4^ Department of Organic Chemistry, Faculty of Chemistry, University of Gdańsk, Gdańsk, Poland; ^5^ Division of Clinical Anatomy, Medical University of Gdańsk, Gdańsk, Poland; ^6^ Department of Physiopathology, Faculty of Medicine, Medical University of Gdańsk, Gdańsk, Poland; ^7^ Department of Biomedical Physics, Faculty of Physics, Adam Mickiewicz University, Poznań, Poland; ^8^ NanoBioMedical Centre, Adam Mickiewicz University, Poznań, Poland; ^9^ Department of Surgical Oncology, Medical University of Gdańsk, Gdańsk, Poland; ^10^ Department of Plastic Surgery, Medical University of Gdańsk, Gdańsk, Poland

**Keywords:** hydrogels, wound healing, skin, RADA16-I, PDGF, peptides, croSEM

## Abstract

**Background:** Wound healing complications affect numerous patients each year, creating significant economic and medical challenges. Currently, available methods are not fully effective in the treatment of chronic or complicated wounds; thus, new methods are constantly sought. Our previous studies showed that a peptide designated as PDGF2 derived from PDGF-BB could be a promising drug candidate for wound treatment and that RADA16-I can serve as a release system for bioactive peptides in wound healing. Based on that, in this work, we designed a new self-assembling hydrogel RADA-PDGF2, connecting both peptides by a sequence specific for neutrophil elastase, and evaluated its activity in wound healing.

**Methods:** The physicochemical properties of the designed scaffold were analyzed using transmission electron microscopy, atomic force microscopy, cryoSEM microscopies, and circular dichroism spectroscopy. The enzymatic cleavage was performed using human neutrophil elastase and monitored using high-performance liquid chromatography and MS spectroscopic techniques. The aforementioned techniques (HPLC and MS) were also used to assess the stability of the peptide in water and human plasma. The biological activity was analyzed on human skin cells using a colorimetric XTT test, collagen synthesis evaluation, and a migration assay. The biocompatibility was analyzed with LDH cytotoxicity assay and flow cytometric analysis of activation of immune cells. Finally, RADA-PDGF2 activity in wound healing was checked in a mouse dorsal skin injury model.

**Results:** The analysis showed that RADA-PDGF2 can self-assemble, form a hydrogel, and release a bioactive sequence when incubated with human elastase. It shows pro-proliferative and pro-migratory properties and accelerates wound closure in the mouse model compared to RADA16-I. In addition, it is not cytotoxic to human cells and does not show immunogenicity. RADA-PDGF2 seems to be a promising drug candidate for wound management.

## 1 Introduction

Chronic wounds are wounds that cannot completely heal in the proper time and manner. They include different types of wounds, e.g., diabetic foot ulcers (DFUs) or venous leg ulcers (VLUs). Nowadays, they constitute an enormous economic burden and a huge challenge for medical staff (Falanga et al., 2022). They are mostly common in elderly patients and can significantly reduce their quality of life. With an ageing society and more frequent incidence of civilization diseases like, e.g., diabetes, the problem of chronic wounds will probably grow (Sen, 2021). On the other hand, currently available methods of wound treatment, which include, e.g., different kinds of wound dressings or cell therapies, are not fully effective, especially in the treatment of complicated, chronic wounds. That is why new methods are constantly being sought.

Platelet-derived growth factor BB (PDGF-BB), one of the first growth factors released into the wound, plays a crucial role in the wound healing process. It is responsible for the formation of granulation tissue, production of extracellular matrix (ECM), takes part in the recruitment of mesenchymal stromal cells (MSCs), and stimulates the proliferation of fibroblasts (White et al., 2021). In 1997, the FDA approved human recombinant PDGF-BB in sodium carboxy-methylcellulose (NaCMC) hydrogel (Regranex^®^) for the treatment of DFUs (Reyes-Martínez et al., 2019). However, because of the high cost of such therapy and the short half-life of intravenously administrated PDGF-BB (a few minutes), it seems that developing new bioactive peptides that mimic its activity and placing it in a carrier which allows its sustained release is a good strategy (Zhang et al., 2021; Jian et al., 2022). Our previous studies on derivatives of PDGF-BB proved that PDGF2 peptide (RLIDRTNANFL) is a promising drug candidate for the stimulation of wound healing. PDGF2 exhibits a favourable safety profile with minimal cytotoxicity and limited immunogenicity *in vitro*, as demonstrated by negligible lymphocyte and basophil activation. Furthermore, it shows strong pro-proliferative properties towards human skin cells, especially keratinocytes, and applied topically in 18% P407 hydrogel accelerates wound healing in a mice model (Deptuła et al., 2020).

In recent years, there has been a big interest in using hydrogels in regenerative medicine. Among other types of hydrogels, self-assembling peptide hydrogels are of great interest in tissue engineering or drug delivery (Levin et al., 2020). Self-assembling peptides are built of short amino acid sequences that can be repeated. They form nanostructures in the assembling process (Lee et al., 2019). Products containing one of them, RADA16-I, are currently used in the clinics. PuraStat^®^ (2.5% v/v aqueous solution) is applied as a surgical hemostatic agent (Gelain et al., 2021). Upon contact with blood, PuraStat^®^ undergoes a pH-induced transformation, forming a hydrogel that serves as a mechanical barrier to control bleeding at the site of application (Subramaniam et al., 2021). In the US, PuraSinus™ (2.5% RADA16-I) is used intraoperatively as a hemostatic wound dressing and an adjunct to wound healing after nasal surgery or trauma, and PuraDerm™ was approved for use as topical wound dressing for treating, e.g., DFUs, surgical wounds or pressure sores (Sankar et al., 2021). RADA16-I can also serve as a carrier for other bioactive compounds. In our previous studies, we developed three biomaterials based on RADA16-I, in which RADA16-I was functionalized with bioactive motifs (GHK, KGK, RDKVYR) by a sequence specific to neutrophil elastase. Designed hybrids had properties similar to RADA16-I, and the linker specific to neutrophil elastase left the bioactive motif free after incubation with the enzyme. Additionally, the obtained materials were safe for human cells and stimulated wound healing *in vivo* in a mouse model (Dzierżyńska et al., 2023). What is more, AAPV was also useful in releasing the same bioactive motifs from a self-assembling peptide fibril (QAGIVV). (Sawicka et al., 2021).

Based on the above-mentioned studies, in this work, we decided to use a similar approach and design a new self-assembling peptide hydrogel named RADA-PDGF2. The molecule is composed of the amino acid sequence of RADA16-I (Ac-RADARADARADARADA-NH_2_) connected with PDGF2 peptide (RLIDRTNANFL) derived from PDGF-BB by the fragment specific for the neutrophil elastase (AAPV). The linker is specific for elastase, an enzyme participating in skin remodeling, and it was introduced to enable the release of the active sequence from the carrier after application in the wound site. The chemical and physicochemical properties of the designed scaffold were analyzed. Its biocompatibility and activity were tested *in vitro* on human skin cells with the use of colorimetric cytotoxicity (LDH) and proliferation (XTT) assay and flow cytometric analysis of activation of human immune cells. In addition, the RADA-PDGF2 effect on skin cell migration and collagen synthesis was evaluated. Finally, a mice dorsal skin injury model was used to analyze their potential to promote wound healing.

## 2 Materials and methods

### 2.1 Synthesis and purification of the RADA-PDGF2 peptide

The synthesis was performed on the CEM Liberty Blue microwave peptide synthesizer on the TentaGel S RAM amide solid support (Rapp Polymere, loading 0.22 mmol/g) utilizing Fmoc/tBu chemistry. The double coupling of the Fmoc/tBu standard protected amino acids was carried out with 5 eq. of amino acid derivative (0.2 M), 5 eq. of DIPCDI (0.5 M in DMF), and 5 eq. of oxyma pure as the racemization suppressor (1 M in DMF) for all cycles. The coupling time and temperature cycles varied depending on the residue, starting with the PDGF2 sequence carried out for 135 s at 90°C each cycle, 360 s at 80°C for the elastase-sensitive fragment, and 10 min +20 min at 80°C for the Ac-(RADA)_4_ moiety. Cycles of Fmoc deprotection were also doubled and carried out for 4 min at room temperature and 5 min at 40°C for the second cycle. The peptide was cleaved from the solid support for 4 h with a cleavage cocktail (92% TFA, 4% TIPSI, and 4%, H_2_O, 5 mL per Gram of resin) and precipitated with cold diethyl ether. The peptides were purified by semi-preparative high-performance liquid chromatography (HPLC) (Shimadzu LC-20AP system). The crude products were dissolved in water and injected into Jupiter Proteo C12 semi-preparative column (Phenomenex, 21.2 mm × 250 mm, 90 Å, and 4 μm). Chromatographic separation was carried out in a linear gradient of 1 → 100% B with eluents as A = 0.1% TFA in water and B = 0.1% TFA, 30% ACN in H_2_O the flow rate of 13 mL/min and ultraviolet (UV) detection at *λ* = 223 nm. After the purification step, the selected fraction was subjected to the exchange of trifluoroacetic counter ions to acetate by semi-preparative HPLC and conditions like the above but with the following solvents instead: A = 10 mM aqueous ammonium acetate (pH 5) and B = 25% ACN in 10 mM aqueous ammonium acetate (pH 5). The purity of the peptide was verified by analytical UHPLC (UltiMate 3000, Dionex) on the Kinetex (Phenomenex) C8 column, 2.6 mm × 100 mm, 100 Å, 2.6 µm in a gradient of 1%–100% B in 15 min (R_t_ = 6.39 min, purity greater than 98%) with 0.5 mL/min flow rate, detection at *λ* = 223 and eluents as A = 0.1% TFA in water and B = 0.1% TFA, 80% ACN in H_2_O. The calculated average molecular weight of the RADA-PDGF2 was confirmed with the MALDI-TOF Biflex III MALDI-TOF mass spectrometer (Bruker Daltonics). The theoretical average molecular weight was 3708.03 Da and obtained m/z of 3708.98 ([M + H]^+^).

### 2.2 Gelation conditions

RADA- PDGF2 gel was created through the dissolution of 10 mg of the peptide in 1 mL of water, resulting in a final concentration of 1%. Prior to a minimum 4-h incubation in a thermal cycler, the peptide was thoroughly mixed. The pH of the resultant hybrid solution was tuned to 5.5. Within the pH range of four to eight, there were no instances of precipitation. pH regulation was achieved using pH strips. For experiments involving enzymatic elastase cleavage, circular dichroism measurement, stability evaluations in water and plasma, as well as XTT and LDH assays, the peptide underwent gelling before being diluted to the appropriate concentration. In the case of AFM and TEM experiments, 1% RADA-PDGF2 gel was obtained by the addition of PBS and subsequently adjusted to the desired concentration. When it came to cryo-SEM measurements and mouse trials, the gel was utilized at a concentration of 10 mg/mL without any further dilution. In order to maintain stability during experiments in water and plasma, with various cell lines, and during mouse studies, the gels were meticulously prepared under sterile conditions.

### 2.3 Peptide stability in water and plasma

Blood samples provided by healthy volunteers were processed with lithium/heparin tubes to eliminate clots by centrifugation. Plasma aliquots were mixed with RADA-PDGF2 peptide for 24 h at 37°C, using a plasma to peptide volume ratio of (4:1% v/v), to obtain final concentration 2.69 µM of RADA-PDGF2 peptide.). Samples were collected at six time points (0, 1, 2, 3, 6, and 24 h) to assess water and plasma stability. The collected samples were then prepared and analyzed according to the protocol described earlier (Dzierżyńska et al., 2023).

### 2.4 Incubation with neutrophil elastase

The enzymatic stability study was performed employing the human neutrophil elastase enzyme (EC No. 3.4.21.37, Sigma Aldrich, USA). The RADA-PDGF2 peptide was dissolved in 25 mM Tris HCl buffer, pH 7.5 to 0.5 mM concentration. The enzyme was added to the peptide and incubated for 30 min at 37°C with agitation and the enzyme: peptide molar ratio of 1:3300. The enzymatic reaction was stopped with 5% aqueous formic acid solution final. The reaction progress was monitored by RP-UHPLC (UltiMate 3000, Dionex) with UV-Vis detector on a Kinetex (Phenomenex) C8 column, 2.6 mm × 100 mm, 100 Å, 2.6 μm in a gradient of 1%–50% B in 15 min and by LC-MS.

### 2.5 Circular dichroism

Peptide molar ellipticity was measured using a Jasco J-815 spectrometer. Hybrid peptide solutions were created at 10 mg/mL, incubated at 37°C for 4 h, and diluted to 0.20 mg/mL. CD spectra were recorded from 25°C to 60°C, with wavelengths ranging from 190 to 260 nm and a scan speed of 2 cm/min. Results represented as mean residue molar ellipticity (MRME) [deg × cm2 × dmol−1] vs. wavelength [nm] were obtained from triplicate measurements. In order to determine the peptides’ denaturation temperatures, the molar ellipticity rotatory angle at a fixed wavelength of 196 nm, [θ]_196_, was measured as a function of temperature (max 100°C). Extrapolation beyond 100°C used GraphPad Prism 8 software with a 4 PL to curve fit. Denaturation temperature was identified as the point of 50% ellipticity change.

### 2.6 Atomic force microscopy

A 1% (v/v) peptide hydrogel solution was prepared and incubated in the PBS buffer. After that, the samples were diluted to a concentration of 0.001%. Samples were applied to a freshly cleaved mica surface and then washed for 30 s with 100 µL of MilliQ water. They were then air-dried for 1 h at room temperature before visualization. A JPK NanoWizard^®^ 4 atomic force microscope was used to analyze the topography of the deposited samples, which were analyzed in QI (Quantitative Imaging) mode using Tap150AL AFM cantilevers from Ted Pella, Inc., Redding, USA. JPK Data Processing software (version spm-6.1.42) was used for further processing and analysis of AFM images.

### 2.7 Transmission electron microscopy

A 1% (v/v) peptide hydrogel was prepared and incubated at 37°C. The obtained hydrogel was then diluted to a concentration of 0.001% (v/v). A small amount of the resulting solution was carefully placed on a microcellulose membrane and stained using a 1.5% (w/v)uranyl acetate solution. Images were taken using a Tecnai Spirit BioTwin FEI transmission electron microscope operating at 120 kV.

### 2.8 CryoSEM

Microscopic images were registered using a JEOL JSM-7001F TTLS scanning electron microscope (JEOL Ltd., Japan) equipped with a PP3000T cryoSEM preparation system, which allows preparation, processing, and transfer of cryo samples into the SEM chamber. The sample was immediately immobilized by rapidly immersing hydrogel droplets (1%) in frozen liquid nitrogen (at about −210°C). It was then transferred under vacuum to a preparation chamber, which was attached to the SEM. In the preparation chamber, the sample was fractured at −185°C to expose the fresh surface. Sublimation followed, and then a thin layer of platinum coating was applied. Finally, the sample was introduced under vacuum into the cryo-stage SEM (−190°C) for surface imaging using an accelerating voltage of 5 kV and a secondary electron detector (SEI).

### 2.9 Fibroblast isolation

Human primary dermal fibroblasts were isolated from skin samples obtained during routine surgical procedures. The procedure was approved by the Independent Bioethics Commission for Research of the Medical University of Gdańsk (NKBBN/387/2014, NKBBN/672/2019). The isolation was performed with the use of an explant culture, with a procedure described before. (Sawicka et al., 2020). Skin samples were cut into fragments and enzymatically digested with dispase (6 units/mL) overnight. Then, the dermis were separated from the epidermis, and the obtained explants were placed in a 6-well plate in DMEM HG medium. The medium was changed every 2 days for 2–3 weeks until the confluence of dermal fibroblasts reached 70%.

### 2.10 Cell culture

Human dermal fibroblasts, 46BR.1N fibroblast (Merck), and HaCaT keratinocytes (Merck) were used in the study. The cells were cultured in DMEM HG medium supplemented with 10% FBS and 1% antibiotics penicillin and streptomycin (P/S) under standard conditions (37°C, 5% CO_2_). HaCaT and 46BR.1N cells were passaged twice a week, while fibroblast once a week. The medium was changed every 2 days.

### 2.11 Analysis of cytotoxicity and cell proliferation

Colorimetric LDH and XTT assays were used to analyze RADA-PDGF2 cytotoxicity and its effect on cell proliferation, respectively. The procedure was performed as previously described, according to the manufacturer’s instructions. (Sawicka et al., 2021). Cells were seeded in 96-well plates in DMEM HG medium with 10% FBS and 1% P/S. The next day, the medium was changed to DMEM HG with 1% P/S (control, test samples) and cell were stimulated by RADA- PDGF2 at concentrations of 0.01–150 μg/mL. For the FBS control, the medium was changed to DMEM HG with 10% FBS and 1% P/S. After 48h, half of the medium was replaced with new and stimulated RADA-PDGF2 again. After 48h and 72 h of incubation, XTT readings were performed. A combination of XTT and PMS reagents was added to each sample, then incubated 4 h and a read at spectrophotometer at 450 nm.

For the LDH assay, the cells were seeded as described above. Cells were stimulated with RADA- PDGF2 at concentrations of 50–150 μg/mL. After 48 h the medium from the wells was transferred to a new plate. LDH reagents mixture was added to the samples for 45 min. A spectrophotometric reading was then taken at 450 nm.

### 2.12 Analysis of cell viability

Cell viability of dermal fibroblast, 46BR.1N fibroblast, and HaCaT keratinocytes was analyzed after 4 days of cell culture on RADA-PDGF2 hydrogel. RADA-PDGF2 was dissolved in water at a concentration of 1% w/v and placed in the inserts (0.4 µm) located in a 24-well plate. The whole was incubated for at least 1 hour at 37°C to allow gelation. Then, twice the volume of DMEM HG medium was added to the gel and left overnight in the incubator. The cells were trypsinized, centrifuge and then seeded on a gel at 50,000 per insert in DMEM HG medium with 2% FBS and 1% P/S. The medium was changed to DMEM HG with 2% FBS and 1% P/S after 2 days of incubation. After 4 days of incubation to assess viability, cells were stained with Calcein-AM (c = 8 µM) and propidium iodide (2 μg/mL) for 30 min and then analyzed by confocal microscopy with the use of Nikon Eclipse Ti-e Confocal Microscope.

### 2.13 Analysis of cell migration

Cell migration was analyzed with the use of ibidi culture inserts. Cells were seeded into insert (20,000 per insert) in DMEM HG medium with 10% FBS and 1% P/S. After 24 h of incubation, cell proliferation was blocked for 2 h with 1 mg/mL mitomycin C. Next, the medium was changed for a fresh serum-free DMEM HG, and RADA-PDGF2 was added. After 24 h of incubation, cells were fixed for 30 min in 4% paraformaldehyde and stained for 15 min with 0.05% crystal violet. Cell migration was analyzed with a Leica DMIL LED inverted microscope and GRAPHAX software. The results are presented as % of untreated control.

### 2.14 Analysis of collagen synthesis by fibroblasts *in vitro*

46BR.1N cells and dermal fibroblasts were seeded into 96-well plates (5,000 cells per well) in DMEM HG medium supplemented with 10% FBS and 1% P/S. After 24 h of incubation in standard conditions, the medium was exchanged for a fresh one, and cells were stimulated with appropriate concentrations of RADA-PDGF2 (10 and 25 μg/mL). Analysis of *in vitro* collagen synthesis was performed after 6 days of incubation with RADA-PDGF2 peptide as previously described. (Sawicka et al., 2021). The medium was removed, and the cells were fixed in Bouin’s fluid for 1 h. The plate was washed for 15 min under running water, dried and stained with Direct Red 80 for 1 h on microplate shaker. Next, the dye was removed, the cells were washed in hydrochloric acid and photographed under a light microscope. The dye was then dissolved in sodium hydroxide for 30 min by shaking. Spectrophotometric reading was performed at 550 nm. The blank was sodium hydroxide. The results are presented as % of untreated control.

### 2.15 Immunological analysis

The analysis of the effect on immune cells *in vitro* was performed with human peripheral blood mononuclear cells (PBMCs) isolated from “buffy coats” using a Ficoll density gradient (Histopaque, Sigma-Aldrich Co.). The procedure was approved by the Independent Bioethics Commission for Research of the Medical University of Gdańsk (NKBBN/387/2014). 100 ul of 1% RADA-PDGF2 was placed in a 24-well plate. Then, culture inserts with a pore diameter of 0.4 μm were placed in the wells, and cells were seeded into them in the amount of 0.5 million cells/0.5 mL RPMI 1640 (P/S, 10% FBS)/well. This was then supplemented with medium alone to a final volume of 1 mL/well. Cells were incubated in standard conditions for 24 h. The negative control was unstimulated cells, grown in the insert, in a well without gels. RADA16-I constituted an additional control. After the incubation, cells were harvested, washed in PBS, counted, and prepared for cytometric analysis. 100,000 cells/100 µL were stained with fluorochrome-conjugated monoclonal antibodies (anti-CD3, anti-CD4, anti-CD8, anti-CD16, anti-CD56, anti-CD25, anti-CD69, anti-CD71, anti-HLA-DR, anti-CD11c, anti-CD80, and anti-CD83). Following 30 min of incubation at room temperature (RT) in the dark, the cells were analyzed using a flow cytometer (LSRFortessa, BD).

### 2.16 Wound healing assay, histological analyses, and quantification of collagen density

The procedures were approved by the Local Ethics Committee for Animal Experimentation in Bydgoszcz, Poland (Approval No. 49/2016) and the experiments were conducted in compliance with the ARRIVE guidelines and in accordance with the European Union Commission Recommendation of 18 June 2007, on guidelines for the accommodation and care of animals used for experimental and other scientific purposes. Eight-week-old mouse females of BALB/c strain were used for experiments. Prior to the experiment, the mice were randomized into treatment and control groups of six each. The mice were anaesthetized with 2%–5% isoflurane; the dorsum’s skin was shaved and disinfected. After the mouse dorsal skin was folded along the spine, two through-and-through symmetrical wounds were made with a 6.0 mm biopsy punch. Immediately after the injury, 10 µL of RADA-PDGF2 peptide hydrogel (1%) was applied to each wound. RADA16-I (the carrier peptide alone), was used as the control. Next, the wounds were covered with a sheet of transparent Tegaderm TM dressing fastened with an adhesive plaster wrapped around the animal’s torso. The peptide application and dressing replacement were repeated for the following 4 days. Then, dressings were replaced every second day until the end of the second week of the experiment. Wounds were photographed with a ruler next to the injury, and wound closures were quantified using an image analysis program ImageJ (Schneider et al., 2012). The edges of the epidermis and dermis restoration were distinguished based on visual observation of photograph documentation.

For histological examination, mice were sacrificed at days 4 and 21 post-injury. After the mice were euthanized, the skin from the back was dissected and stored in formalin. The skin samples were then embedded in paraffin, cut into 5-µm sections, and stained with hematoxylin-eosin, or Masson’s trichrome. The stained sections were examined using a Leica DM IL microscope. Collagen density in skin wounds was estimated by the method described before (Ruifrok and Johnston, 2001), using ImageJ software with a color deconvolution plug-in. seven to nine images representing two to three mice of Masson-Trichrome stained skin sections were included for each estimation.

### 2.17 Statistical analysis

Statistical significance was determined with the Mann-Whitney *U*-test (*p* < 0.05) using STATISTICA software (StatSoft Polska, Kraków, Poland) and XLSTAT (Addinsoft). Graphs were plotted with GraphPad Prism 5 software.

## 3 Results

### 3.1 Peptide design

**FIGURE 1:**
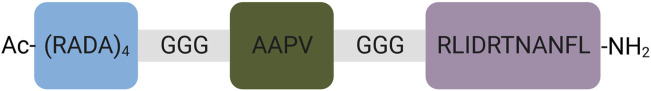
General scheme of the designed scaffolding amino-acid sequence. The scaffold consists of 4 elements: gel-forming (blue), neutrophil elastase-sensitive (green), biologically active (pink), and linkers (grey). Created with BioRender.com.

The main objective of our research was to obtain a multifunctional peptide hydrogel with defined biological activity and a controlled system of its release (active sequence) into the wound environment. The designed peptide hybrid is expected not only to provide structural support but also to stimulate angiogenesis, attract immune and endothelial cells to the site of injury, and increase fibroblast proliferation and migration. The designed peptide, called RADA-PDGF2, is a combination of 4 different amino acid sequences with distinct functionality. The first sequence is the gel-forming sequence, situated at the *N-*terminus of the designed peptide, which is formed by a quadruple sequence Arg-Ala-Asp-Ala, known in the literature as RADA16-I (Zhang, 2017). Next, a glycine linker is present in the peptide sequence. This linker occurs on both sides of the third component - a sequence specific for neutrophil elastase activity (Ala-Ala-Pro-Val). The glycine linkers were inserted to facilitate the enzyme’s access to the sequence which is prone to be hydrolyzed by the enzyme. Glycine is a small amino acid with no extended functional groups in the side chains, which increases the flexibility of this peptide fragment and is likely to facilitate the enzyme to access its specific sequence. The last, fourth sequence located at the *C-*terminus of the peptide represents a sequence with proven biological activity. The active sequence used in this study is the PDGF2 peptide Arg-Leu-Ile-Asp-Arg-Thr-Asn-Ala-Asn-Phe-Leu, known for its wound-healing-accelerating properties (Deptuła et al., 2020). The designed RADA-PDGF2 peptide sequence is shown in the schematic diagram in Figure 1.

The release of PDGF2 peptide from the designed scaffold is based on the enzymatic activity of human neutrophil elastase, a catalyst that cleaves hybrid peptides at a specific site designated AAPV. This cleavage activity is responsible for initiating the controlled release of active compounds. The choice of this enzyme is targeted because neutrophil elastase is commonly found in wounds, and its concentration is increased in infected wound samples, consistent with the context of wound healing (Wilgus et al., 2013). To the best of our prior knowledge, it has been established that peptides prepared using this approach effectively retain their intended functionality (Sawicka et al., 2021; Dzierżyńska et al., 2023). Specifically, these peptides exhibit the ability to form a 1% hydrogel in an aqueous environment and then release the active peptide fragment upon exposure to neutrophil elastase.

### 3.2 Peptide stability in water and human plasma

**FIGURE 2:**
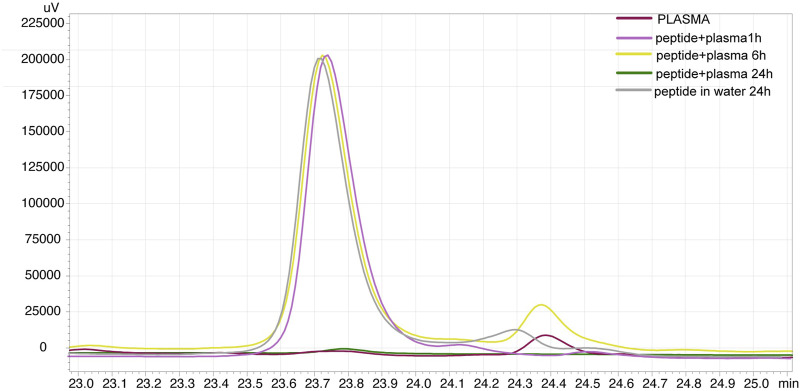
Stability analysis of RADA-PDGF2 peptide in human plasma over time. In the chromatogram slice, it is evident that the RADA-PDGF2 peptide maintains its chemical integrity and remains stable for the initial 6-h period. After this time, the peak loss can be seen on the chromatogram after 24 h of incubation (green line), indicating complete hydrolysis of the peptide. This observation highlights the time-dependent degradation profile of RADA-PDGF2 in plasma.

The next step verified whether the designed RADA-PDGF2 is stable enough to create a stable chemical peptide hydrogel. This information is critical because it determines the rest of the planned experiments and the possibility of using the peptide in a long-term application. In the aqueous solution, the RADA-PDGF2 peptide exhibited chemical stability over 24 h. HPLC analysis revealed no significant alterations in peptide peak profiles, indicating that the peptide retained its chemical properties in water. Conversely, stability testing in human plasma yielded contrasting results (Figure 2). The RADA-PDGF2 peptide displayed initial stability, with no apparent degradation evident for up to 6 h, as shown in Figure 2 (yellow line). However, prolonged incubation for 24 h led to a complete hydrolysis of the peptide, as indicated by the disappearance of the intact peptide peak in the HPLC chromatogram, as shown in Figure 2 (green line).

### 3.3 Neutrophil elastase cleavage activity

**FIGURE 3:**
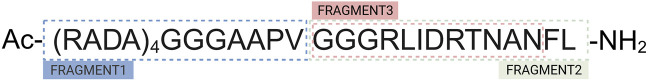
The fragments of RADA-PDGF2 peptide after enzymatic digestion. The figure clarifies the different locations in the peptide sequence where hydrolysis occurs, yielding identifiable fragments. Created with BioRender.com.

In the next step, we decided to verify whether the designed RADA-PDGF2 peptide hydrolyzes at the designated site, i.e., in the region of the sequence susceptible to neutrophil elastase AAPV. The digestion was tracked by UHPLC, and the released fragments were identified by mass spectrometry. The truncates analysis revealed that the proteolytic sites for the elastase are the most susceptible in the region of the elastase-specific sequence, predominantly after the valine residue (Figure 3, Fragment 1). However, the fragment lacking the last two *C*-terminal amino acids was also detectable as minor (Figure 1, Fragment 3). The scheme of cleavage sites is presented in Figure 3 and the fragments are listed in Supplementary Table S1 (Supplementary Material) Notably, the degradation pattern depicts a consistent cleavage site that leads to the release of fragments encompassing the bioactive PDGF2 peptide (Figure 1, Fragment 2).

### 3.4 Structural characterization of RADA-PDGF2

**FIGURE 4:**
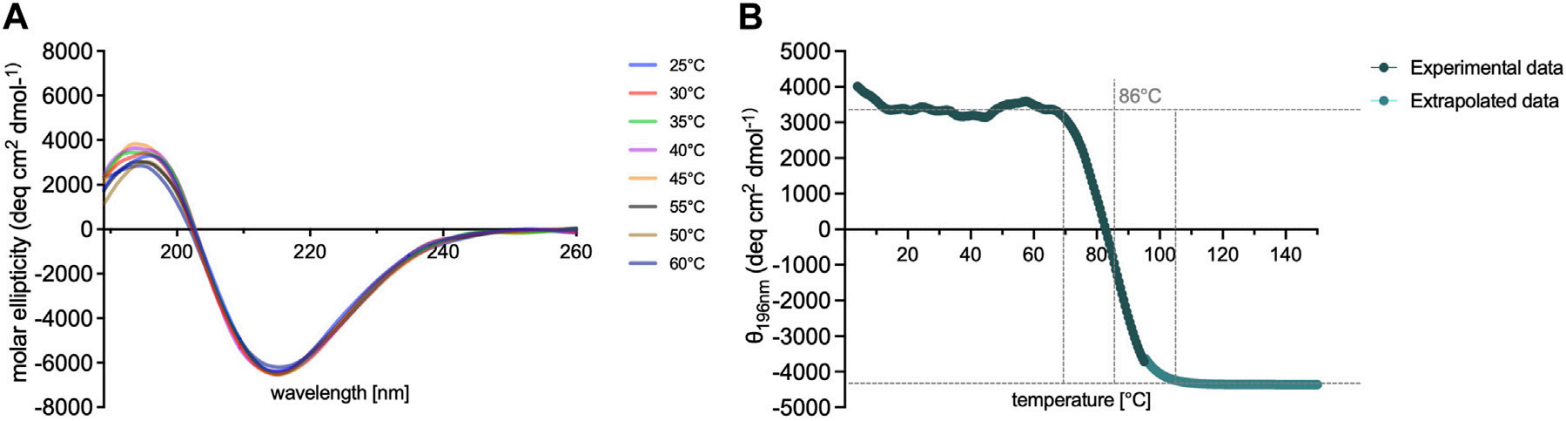
Spectroscopic analysis of RADA-PDGF2 peptide. Graphs **(A, B)** illustrates the relationship between temperature (ranging from 4°C to 95°C) and the CD **(B)** signal showcasing the response of the peptide’s ordered *β*-sheet conformation. Throughout the examined temperature range, the RADA-PDGF2 peptide exhibits remarkable conformational stability, with a well-defined *β*-sheet structure being retained.

The CD spectra exhibited distinct bands indicating the arrangement of the *β*-sheet secondary structure of the peptide. Notably, a well-defined maximum at approximately 196 nm and a corresponding minimum at 214 nm was observed (Figure 4A). These spectral features are characteristic of the *β*-sheet conformation, highlighting the propensity of the peptide to adopt a stable secondary structure at a broad range of temperatures (25°C-60°C).

### 3.5 Thermal stability of RADA-PDGF2

To further assess the thermal stability of the RADA-PDGF2 hydrogel. For this purpose CD spectra were performed at a fixed wavelength of 196 nm across an extended temperature gradient ranging from 4°C to 95°C. Figure 4B illustrates the observed trend, depicting the thermal behavior of the peptide. The RADA-PDGF2 peptide displayed sustained conformational stability up to 86°C, beyond which the ordered structure of the hydrogel began to progressively lose its arrangement. This thermal denaturation phenomenon, marked by the loss of characteristic *β*-sheet bands, suggests a transition from a well-defined gelling secondary structure to a more disordered and non-gelling state.

### 3.6 Characterization of RADA-PDGF2 peptide hydrogel using microscopic techniques

In this study, a comprehensive study was conducted to elucidate the structural characteristics of the RADA-PDGF2 peptide fibers. To achieve this, a combination of advanced microscopic techniques was used, including atomic force microscopy (AFM), transmission electron microscopy (TEM), and scanning cryoSEM microscopy. Each of these methodologies was used to capture distinct viewpoints of the peptide’s architecture, offering independent and highly complementary insights that collectively contribute to understanding the evolving structures. AFM provided high-resolution topographical images of the peptide fibers, enabling a detailed examination of their surface morphology. TEM was selected to visualize the internal structure of the fibers at a nanometer scale, revealing intricate details of their arrangement and organization. Additionally, scanning cryoSEM microscopy offered a freeze-fracture approach, preserving the native state of the hydrogel structures and providing a unique perspective on their three-dimensional arrangement.

**FIGURE 5:**
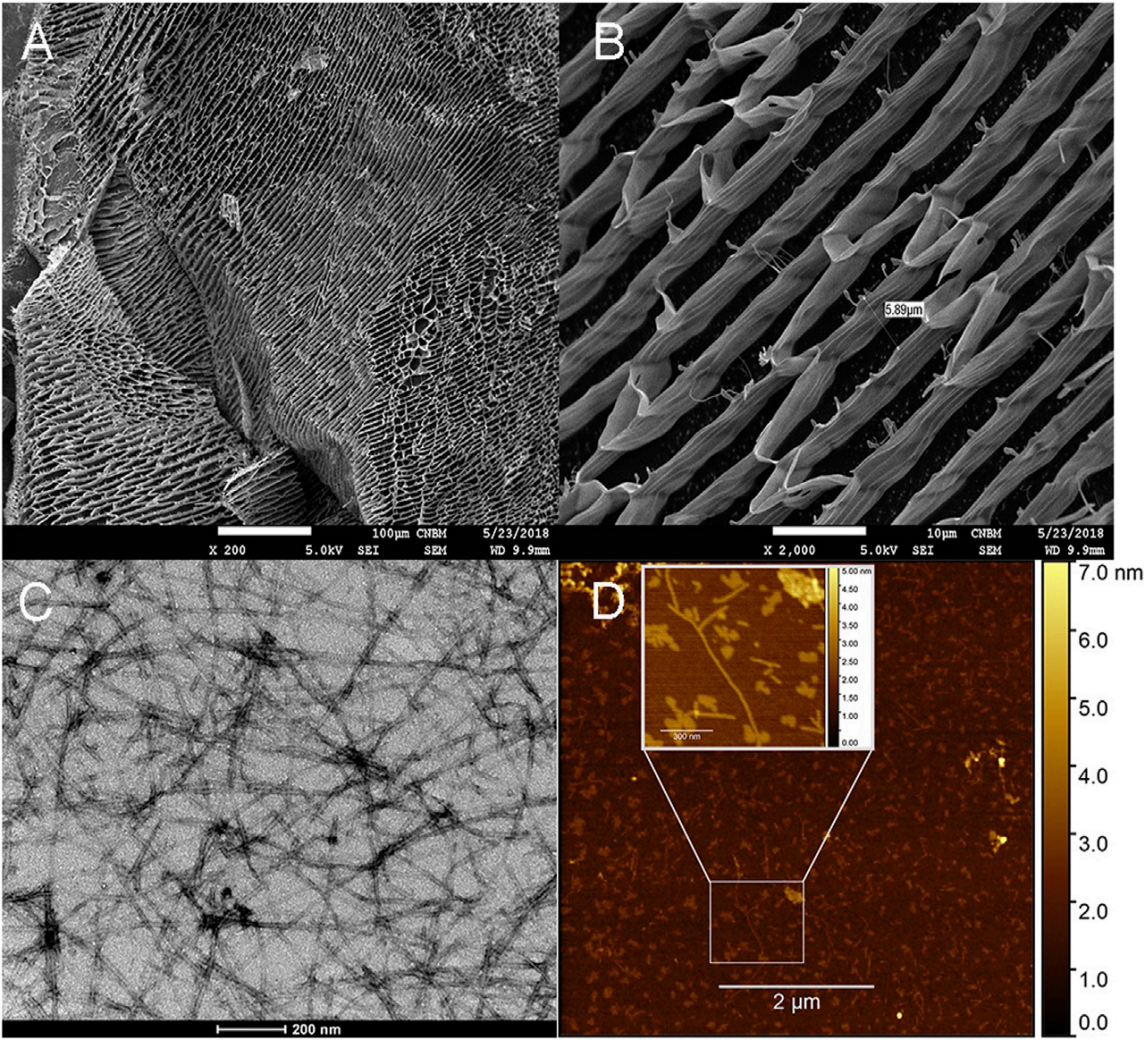
Microstructure and nanoscale characterization of 1% RADA-PDGF2 hydrogel and aqueous solutions. CryoSEM images for 1% RADA-PDGF2 hydrogel **(A, B)** aqueous solutions imaged using TEM microscopy **(C)** and AFM **(D)**.

The results of the experiment indicate that the RADA-PDGF2 peptide when in aqueous solutions, forms a network of fibers with a length of 200–600 nm and a width of 4–7 nm, as observed through TEM and AFM images (Figures 5C, D). Based on AFM images the approximate diameter of the investigated fibers is 3 nm. Observed fibers are distributed isotropically on the mica surface, exhibit a small tendency to association and formation of small aggregates. Furthermore, the cryoSEM images obtained from a 1% peptide hydrogel showcase a highly organized structure resulting from the self-assembly of the peptide (Figure 5A). This structured arrangement becomes clearly visible at lower magnifications and exhibits characteristics resembling a honeycomb-like pattern. The advanced magnification capability of cryoSEM images facilitates accurate measurement of the inter-unit distances within the structure. In this case, the measured distance between the blocks of successive units is approximately 6 μm and the length of the blocks intended ranges from 25 to 70 μm (Figure 5B). When comparing these findings to the available literature on the RADA16-I (PuraMatrix) peptide, noteworthy parallels are evident. Similar to RADA-PDGF2, RADA16-I has been recognized to form fibrous networks in aqueous solutions, showcasing analogous self-assembly tendencies (Sun et al., 2016). The strikingly ordered structure observed in the cryoSEM images aligns with the known structural organization of RADA16-I hydrogels.

### 3.7 Cytotoxicity, cell proliferation, and viability

Analysis of potential cytotoxicity of designed compounds or scaffolds is crucial to eliminating toxic substances in the early stages of their design. For new wound healing candidates, evaluating their effect on the viability and proliferation of skin cells is also essential. In this study, cytotoxicity analysis and the effect on proliferation were performed in concentrations that do not undergo the gelation process using colourimetric methods. Additionally, analysis of cells seeded on the RADA-PDGF2 hydrogel was performed with confocal microscopy.

**FIGURE 6:**
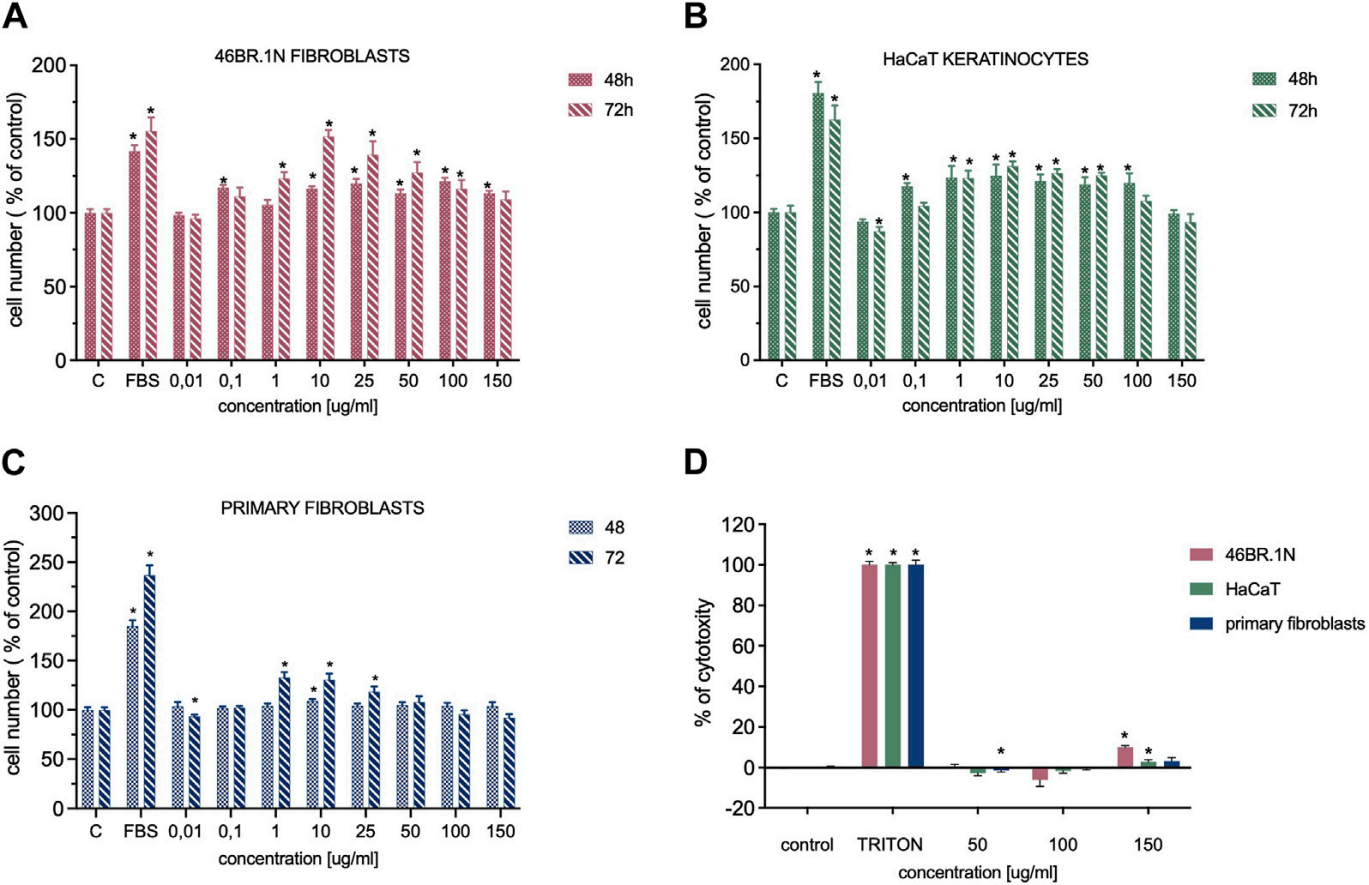
Effect of RADA-PDGF2 on the proliferation **(A)** of 46BR.1N fibroblasts, **(B)** HaCaT keratinocytes, **(C)** primary fibroblast and its cytotoxicity **(D)** towards these cells. The graphs represent the mean ± SEM of 3 independent experiments (4 replicates in each, *n* = 12). * - statistically significant differences compared to controls (cells grown in serum-free medium), Mann -Whitney *U*-test, *p* < 0.05.

The results showed that the RADA-PDGF2 peptide stimulates proliferation of 46BR.1N fibroblast - a 10%–20% increase in proliferation relative to controls was visible in concentrations of 0.1; 10–150 μg/mL after 48h and 20%–60% concentrations of 1.0–100 μg/mL after 72 h of incubation (Figure 6A). A 20%–30% increase in proliferation was observed for HaCaT keratinocytes at 0.1–100 μg/mL after 48 h and 1.0–50 μg/mL after 72 h of incubation (Figure 6B). For human primary dermal fibroblasts, a pro-proliferative effect was observed only after 72 h of incubation–a 20%–30% increase compared to untreated control in concentrations of 1–25 μg/mL (Figure 6C). RADA-PDGF2 does not significantly inhibit the proliferation of tested cells, but at a concentration of 150 μg/mL, it shows slight (about 10%) cytotoxicity to 46BR.1N cells (Figure 6D).

**FIGURE 7:**
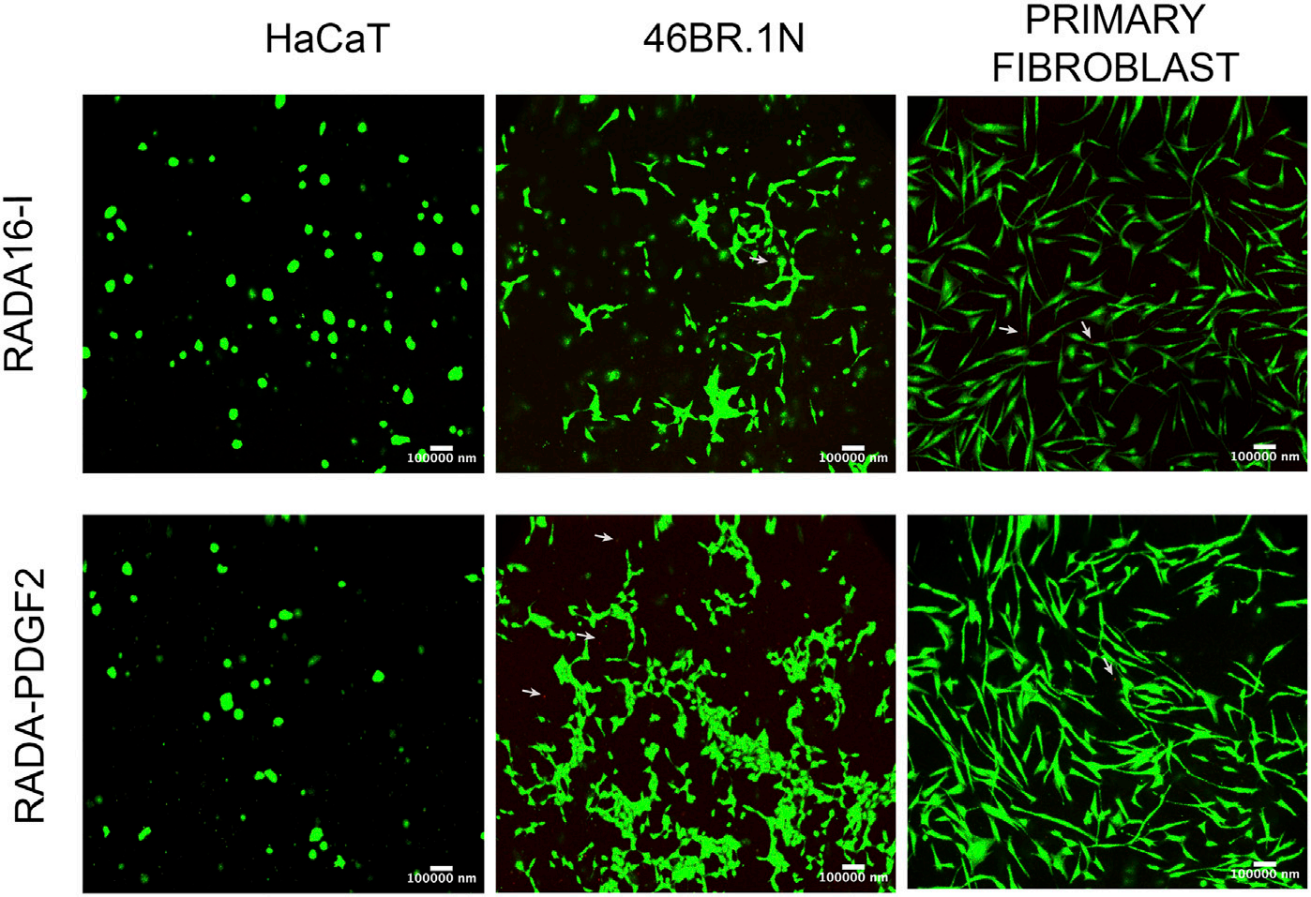
Viability of human keratinocytes (HaCaT), fibroblasts (46BR.1N), and primary fibroblasts after 4 days of cell culture on RADA-PDGF2 hydrogel. Human skin cells were stained with calcein-AM and propidium iodide to visualize live (green) and dead (red) cells indicated by white arrows.

Additionally, skin cell viability was checked after 4 days of incubation on RADA-PDGF2 hydrogel (Figure 7). Cells were stained with calcein-AM (live cells) and propidium iodide (dead cells, indicated with white arrows). The analysis showed that the cells remained viable after 4 days of cell culture on RADA-PDGF2. Only a few dead cells were visible in the field of view. The cells looked similar to the control cells cultured on RADA16-I.

### 3.8 Cell migration and collagen synthesis

Both cell migration and collagen synthesis are important elements of the wound healing process. Also, collagen synthesis by skin fibroblasts is crucial for proper scar formation. That is why, in the study, we have decided to analyze the effect of RADA-PDGF2 (10 and 25 μg/mL) on the migration of skin cells and collagen synthesis by human dermal fibroblasts.

**FIGURE 8:**
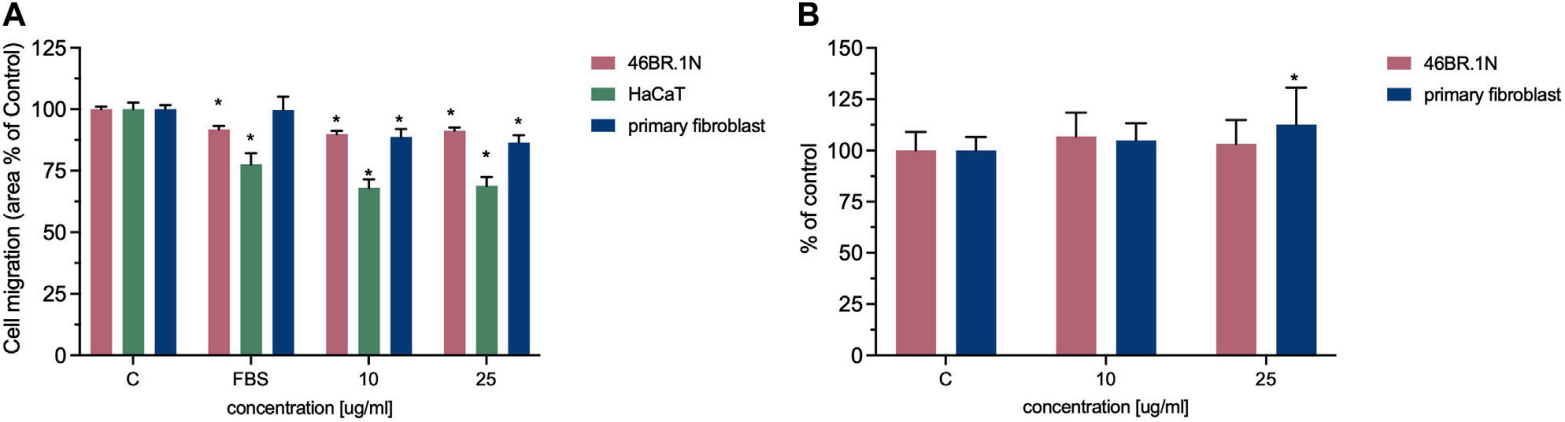
Effect of RADA-PDGF2 on the **(A)** migration of 46BR.1N fibroblasts, HaCaT keratinocytes, primary fibroblast, and **(B)** collagen synthesis by human fibroblasts. The graphs represent the mean ± SEM of 3 independent experiments (4 replicates in each, *n* = 12). * - statistically significant differences compared to controls (cells grown in serum-free medium), Mann -Whitney *U*-test, *p* < 0.05.

The obtained results show that RADA-PDGF2 stimulates the migration of human skin cells. The most potent effect was observed for HaCaT keratinocytes–about a 30% increase in cell migration compared to untreated control in both tested concentrations. A smaller pro-migration effect was observed for fibroblasts - about 10% and 12%–15% in both concentrations for the 46BR.1N cell line and primary fibroblasts, respectively (Figure 8A; Supplementary Figure S2). The analysis of collagen synthesis showed about a 10% increase for human dermal primary fibroblast stimulated with 25 μg/mL RADA-PDGF2 (Figure 8B).

### 3.9 Immunogenicity

In the context of biocompatibility of newly designed scaffolds, it is important to analyze their potential immunogenicity, e.g., by analyzing their effect on the immune cells *in vitro*. Studies on the effect of RADA-PDGF2 and RADA16-I (control) scaffolds on immune cells were performed using isolated peripheral blood mononuclear cells (PBMCs). PBMCs were incubated in culture inserts in the presence of tested RADA-PDGF2 (1%) for 24 h; then, the effect was analyzed by flow cytometry. The extent of T cell (CD3/CD4/CD8) and NK (CD16/CD56) stimulation was determined by assessing the expression of CD69, CD71, CD25, and HLA- DR activation markers. The activation of dendritic cells (DCs) was also examined (inactive phenotype: CD11^+^CD80^−^, CD11^+^CD83^−^, active: CD11^+^CD80^+^, CD11^+^, CD83^+^).

**FIGURE 9:**
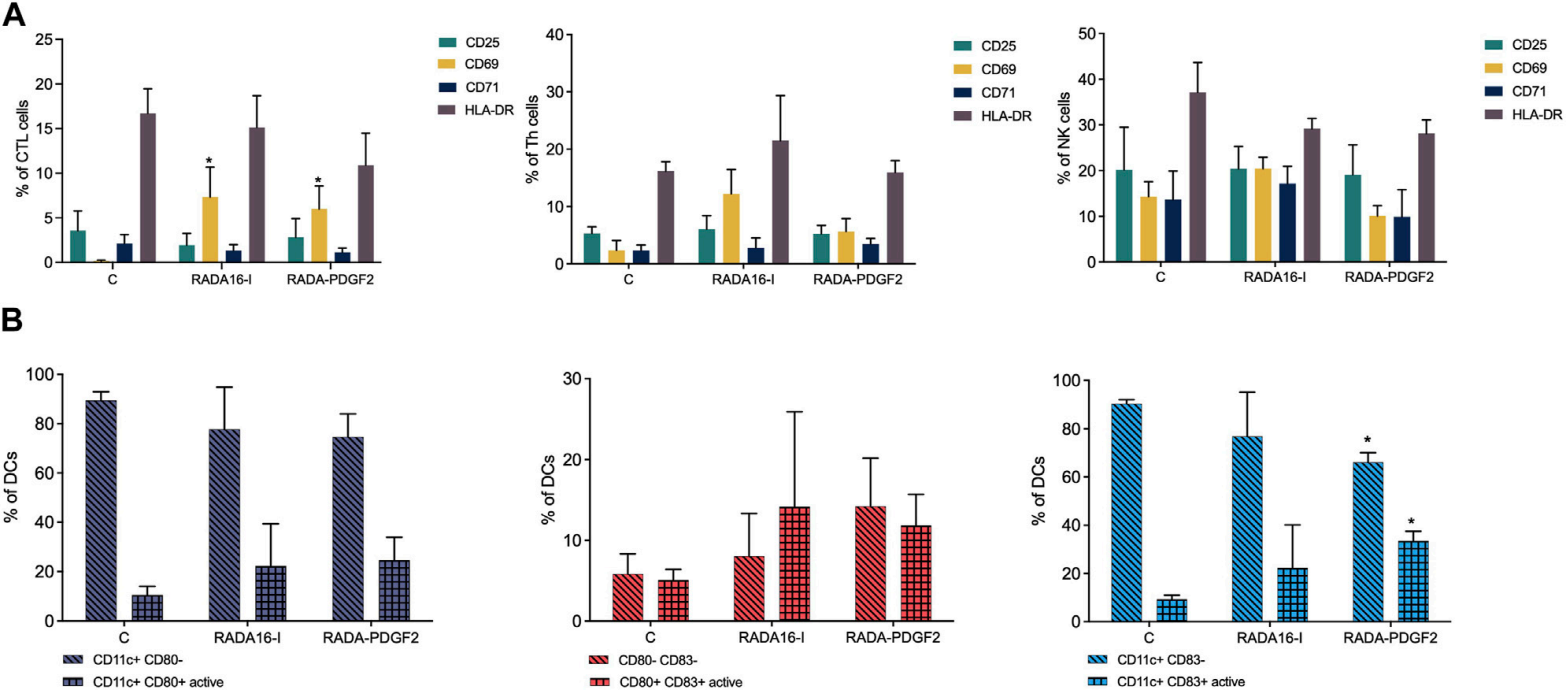
Analysis of the effect of RADA-PDGF2 on immune cells after 24 h of incubation. **(A)** Expression level of surface activation markers (CD25, CD69, CD71, HLA-DR) in individual cell subpopulations (CTL, Th, NK) after incubation in the presence of RADA-PDGF2. **(B)** Activation of dendritic cells after incubation with RADA-PDGF2. The graphs represent the mean ± SEM of 3 independent experiments.

For Th cells, after 24 h of incubation, any statistically significant differences between unstimulated cells and cells stimulated with RADA-PDGF2 were not observed. Similarly, no statistical significance was noted for the NK cells, but a slight increase in the expression of CD25 and a reduction in the expression of CD71 were observed. An increased expression of CD69 marker characterized cytotoxic lymphocytes (CTL) after 24 h of incubation, but a similar effect was obtained for RADA16-I (Figure 9A). Due to the low values of the above changes, the tested RADA-PDGF2 and RADA16-I peptides can be considered safe in an immunological context.

Interestingly, after 24 h of incubation, a statistically significant increase in the CD11c^+^CD83^+^ active DCs population was observed for PBMCs stimulated with RADA-PDGF2 compared to untreated control (Figure 9B).

### 3.10 Murine dorsal skin excision wound model

A dorsal skin excision model was used to assess the effect of RADA-PDGF2 (1%) hydrogel on wound healing. RADA16-I peptide was used for comparison (Dzierżyńska et al., 2023). The excess of skin wound closure involving both the dermis and epidermis and that of epithelialization alone were analyzed at days 0, 2, 4, 7, 9, 11, 14, and 18 post-injury. In addition, collagen density in histological samples from day 18 post-injury and the thickness of epithelial membranes formed at day 4 post-injury were quantified. The experiments were performed using females of BALB/c mice, 8-week-olds, on the initial day of the experiment.

**FIGURE 10:**
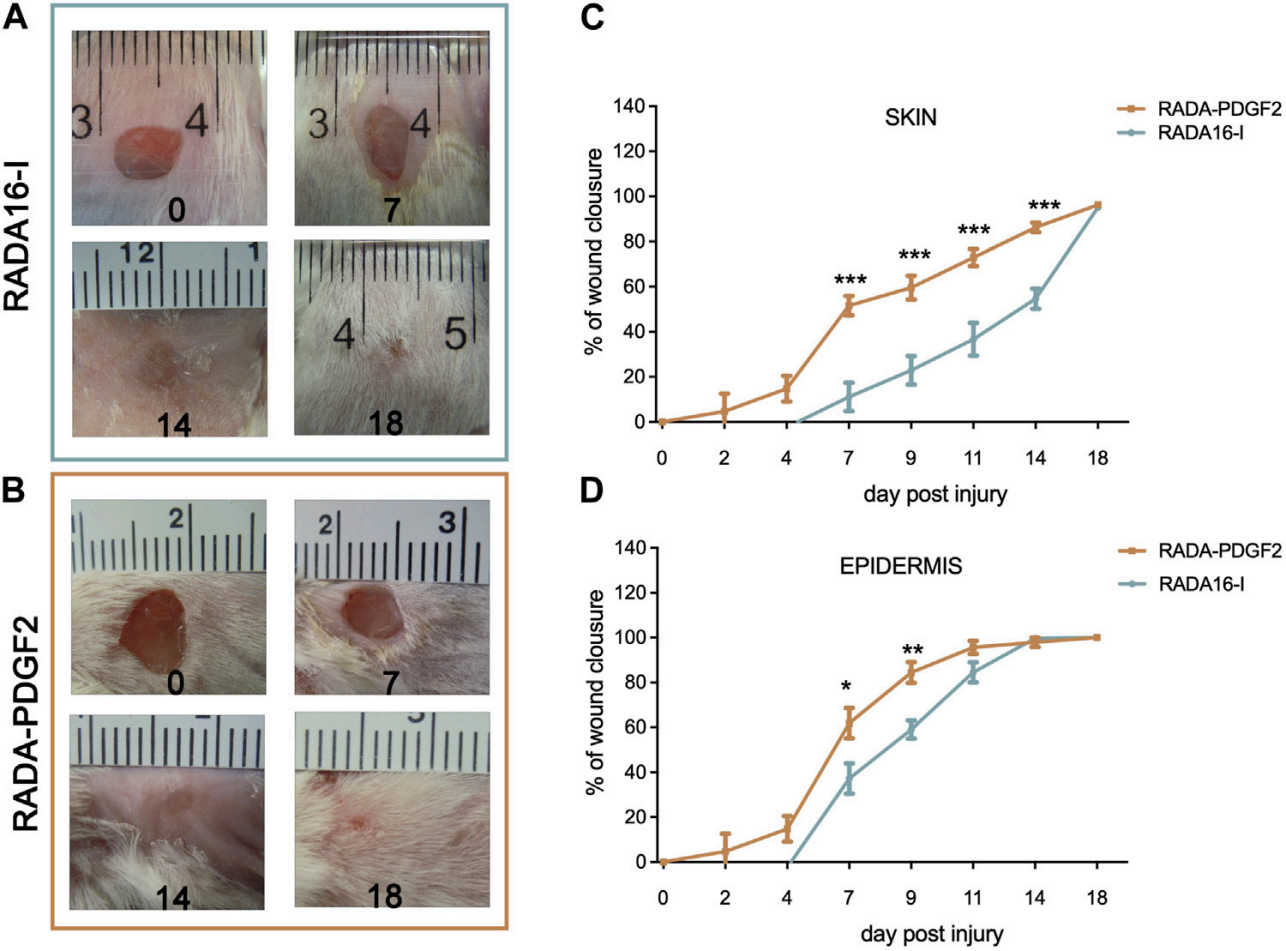
The healing process in skin wounds treated with RADA16-I and RADA-PDGF2 hybrid hydrogels. Representative photographs of the wound healing process on days 0, 7, 14, and 18 post-injury for **(A)** RADA16-I and **(B)** RADA-PDGF2; **(C)** the mean area of full-thickness wound closure throughout the experiment; **(D)**-the mean area of wound epithelialization throughout the experiment The graphs represent the mean ± SEM. Statistically significant differences, determined by the Mann-Whitney *U* test, are marked with asterisks as follows: * - *p* < 0.05; ** - *p* < 0.01; *** - *p* < 0.001, *n* = 12 (number of wounds).

For the RADA-PDGF2 group, a statistically significant reduction in the wound area compared to the control group receiving RADA16-I was observed from the seventh day after the injury, reaching 60% mean closure on day 9 compared to 23% in the control group (Figure 10C). On day 18, complete closure was observed in the treatment and control groups, and a small scar was formed (Figures 10A, B). The rate of epithelialization was significantly accelerated in RADA-PDGF2 compared to the control. On day 7 post-injury, 54% of the initial wound area was covered with epidermis in RADA-PDGF2-treated mice, contrasted to 35% in the control group. On day 14, almost all wounds in both groups were covered with epidermis (Figure 10D).

**FIGURE 11:**
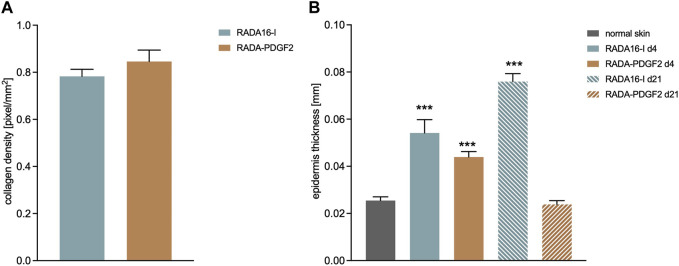
Collagen density on day 21 post-injury **(A)** and the thickness of epidermis on day 4 and 21 post-injury in RADA-treated wounds compared to uninjured skin **(B)**.***- statistically significant differences compared to normal skin, U-Mann Whitney test, *p* < 0.001.

**FIGURE 12:**
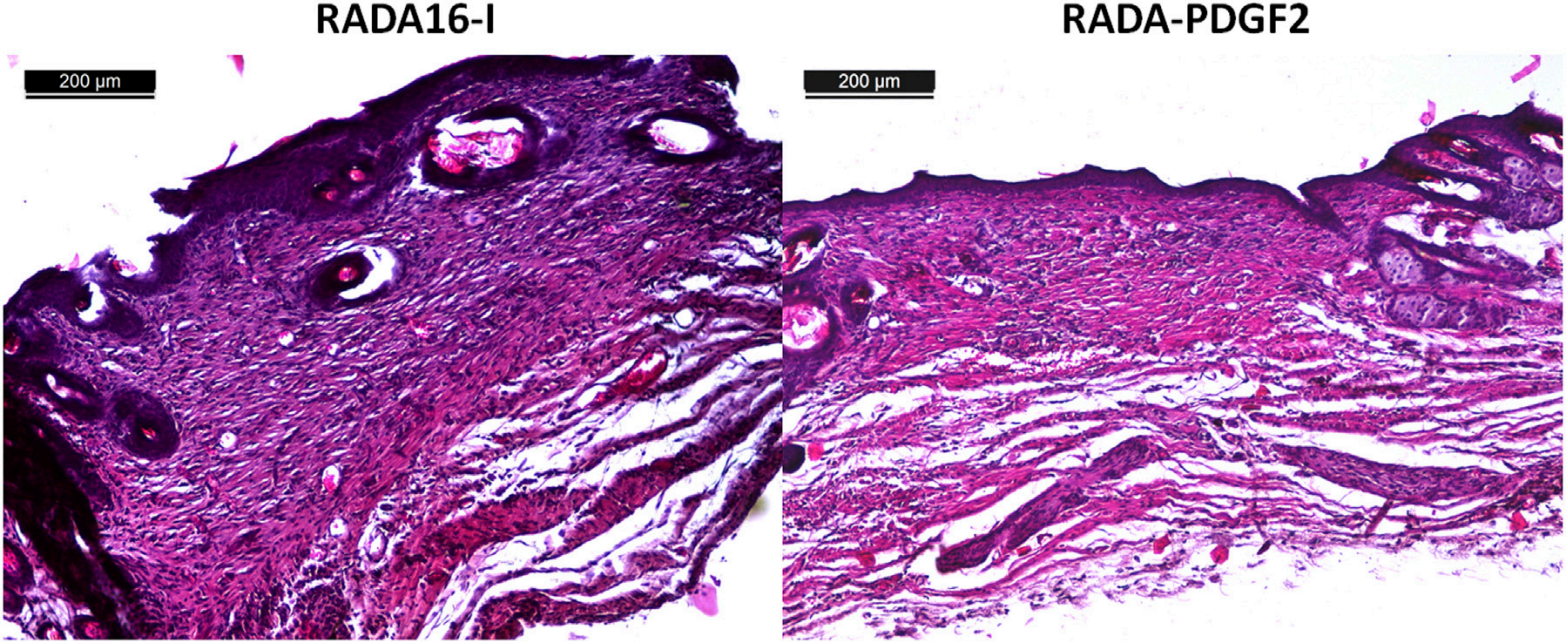
Histological examination of mouse sections of mice from the control (RADA16-I) and RADA-PDGF2 groups, stained with hematoxylin and eosin on day 21 post-injury.

Collagen density was examined in the restored skin on day 21 post-injury. The RADA-PDGF2-treated skin had a slightly higher collagen density in the wound area than the RADA16-I-treated one, but the difference was not statistically significant (Figure 11A). The epidermis thickness examined on day 4 post-injury increased significantly in both RADA-PDGF2 and RADA16-I treated wounds compared to the uninjured skin; on day 21 post-injury, the epidermis remained thicker in RADA 16-I but not in RADA-PDGF2-treated mice, where it decreased to the levels determined in uninjured skin (Figure 11B). Figure 12 shows representative images of tissue architecture in the wound area on day 21 post-injury.

## 4 Discussion

Chronic wounds have emerged as a significant challenge in modern healthcare, exerting a profound impact on both patient quality of life and healthcare expenditure. These persistent wounds encompass a spectrum of conditions, such as diabetic foot ulcers (DFUs) and venous leg ulcers (VLUs), with a heightened prevalence among elderly individuals and those afflicted by diabetes. Despite the existence of various treatment modalities, including wound dressings and cell therapies, the management of complex chronic wounds remains largely unsatisfactory. With an aging population and an escalating incidence of diabetes, the burden of chronic wounds is poised to rise dramatically, necessitating innovative approaches to wound healing. Platelet-derived growth factor-BB (PDGF-BB) holds a pivotal role in the wound healing process. Notwithstanding the approval of recombinant PDGF-BB therapies, challenges such as exorbitant costs and limited half-lives have ignited interest in the development of bioactive peptides that mimic PDGF-BB activity while allowing for sustained release. Prior investigations have unveiled the potential of the PDGF2 peptide (RLIDRTNANFL) as a wound healing stimulant, showcasing attributes like low cytotoxicity, the promotion of cell proliferation, and when applied topically, the acceleration of wound healing in a murine model (Deptuła et al., 2020). The use of self-assembling peptide hydrogels has garnered substantial attention in regenerative medicine due to their adaptable properties and versatility. RADA16-I, an exemplary self-assembling peptide, has already found clinical applications as a surgical hemostatic agent, wound dressing, and tissue engineering scaffold (Sankar et al., 2021). Building upon the promise of self-assembling peptide hydrogels, we have conceived a novel scaffold denominated RADA-PDGF2. This hybrid construct incorporates the RADA16-I sequence linked with the PDGF2 peptide via a neutrophil elastase-specific fragment (AAPV). This unique linker facilitates the isolation of the active sequence post-application, ensuring its sustained bioactivity.

In this study, we comprehensively characterize the chemical and physicochemical attributes of RADA-PDGF2 and assess its biocompatibility, bioactivity, impact on cell migration, and collagen synthesis *in vitro* using human skin cells. Furthermore, we performed a murine dorsal skin injury model to investigate the potential of RADA-PDGF2 in promoting wound healing. Through these investigations, we aim to contribute valuable insights into the development of innovative wound healing strategies to address the growing challenge of chronic wounds in modern healthcare.

The stability of peptides is a critical consideration when designing peptide-based biomaterials for tissue engineering and regenerative medicine (Al Musaimi et al., 2022). In this study, we investigated the stability of the RADA-PDGF2 peptide, focusing on its potential to form a stable chemical peptide hydrogel as well as its suitability for long-term applications. The stability assessment of the RADA-PDGF2 peptide was conducted in two distinct environments: aqueous solution and human plasma. In the aqueous solution, our results indicated that the RADA-PDGF2 peptide exhibited good chemical stability over 24 h. The absence of significant alterations in the peptide peak profiles observed through HPLC analysis further confirms the peptide’s ability to retain its chemical properties in water. Subsequently, we performed studies to assess the stability of the RADA-PDGF2 peptide in a more complex physiological environment, represented by human plasma. During the initial 6-h incubation period, we observed no noticeable peptide degradation, suggesting an initial resistance to enzymatic hydrolysis. This initial stability is consistent with the potential for short-term applications or interventions. Nevertheless, an extended incubation period of 24 h in human plasma resulted in significant and complete disappearance of the RADA-PDGF2 peptide peak on the HPLC chromatogram, as shown in Figure 2. This disappearance may occur due to peptide degradation by enzymes present in plasma (Jenssen and Aspmo, 2008), as well as by interactions with albumins, which have diversity of functions including transport and binding of different molecules (Rondeau and Bourdon, 2011). Both our previous stability studies for the RADA16-I peptide (Dzierżyńska et al., 2023) and the PDGF2 active sequence (Rodziewicz-Motowidło et al., 2022) did not show prolonged presence of peptide in plasma, so the stability of 6 h is surprising. Perhaps the formation of the RADA-PDGF2 hybrid leads to different intermolecular interactions that protect the peptide from rapid degradation. To this end, further studies including molecular modeling are needed. This initial stability suggests that the RADA-PDGF2 peptide may have unique structural characteristics. For this reason, the results obtained indicating more than 6 h stability of RADA-PDGF2 hybrid is a very promising result.

Subsequently, we tested whether the designed RADA-PDGF2 hydrogel is capable of releasing free PDGF2 peptide while exposed to human neutrophil elastase. This aspect was a key to this research. The analysis of the RADA-PDGF2 peptide digestion revealed that the proteolysis by elastase is located in the elastase-specific sequence region, which is AAPV. By incubating the RADA-PDGF2 peptide in the presence of elastase, we were able to confirm that the release of the entire PDGF2 sequence from the RADA-PDGF2 hybrid occurred. Due to this experimental result, we can conclude that the carrier we designed is stable in human plasma and, in addition, plays its role as a storage for substances with biological effects. However, it is worth noting that a smaller fragment lacking the last two amino acids at the C-terminus was also detectable.

Circular dichroism analysis was employed as a powerful tool to delve into the secondary structure of the RADA-PDGF2 peptide across a spectrum of temperatures. CD spectra of RADA-PDGF2 are emblematic of the *β*-sheet conformation, indicating the peptide’s propensity to maintain a stable secondary structure during the hydrogel formation process. (Gelain et al., 2020). Analysis of literature data showed that other peptide analogs based on the RADA16-I sequence exhibit very similar circular dichroism spectra. However, modification of the RADA16-I sequence by elongating the amino acid chain may affect the disruption of the self-assembly process and thus reduce the intensity of the formed secondary structures visible on the CD spectrum (Wang et al., 2011; Han et al., 2022).

The thermal stability results show extremely high-temperature stability of the RADA-PDGF2 peptide. This revelation highlights the potential utility of the RADA-PDGF2 peptide hydrogel in applications that demand stability at physiological temperatures. However, the observed thermal destabilization beyond 86°C underscores the importance of considering the thermal conditions when designing hydrogel-based systems for specific applications. In that case, the hydrogel cannot be autoclaved after preparation, but it must be prepared in sterile conditions before the hydrogelation process. These findings contribute to the broader understanding of the RADA-PDGF2 peptide’s behavior and its potential utility in biotechnological and biomedical contexts. The observed conformational stability within defined thermal limits offers a foundation for further research aimed at optimizing the peptide’s performance of peptides as a biomaterials and exploring their applicability in various therapeutic modalities. Other analogs of the RADA16-I peptide show lower temperature stability than the RADA-PDGF2 peptide. In comparison, RADA16-I peptides with an additional GPGGY or GGAGGS sequence retain structural stability up to 50°C. Above this temperature, there is either a complete disruption or a drastic reduction of the *β*-sheet structure (Sun and Zhao, 2012).

In the next research stage, we aimed, we aimed to discover the structural features of RADA-PDGF2 peptide fibers. To achieve a complex understanding of these structures, we used a synergistic approach, integrating advanced microscopic techniques: AFM, TEM, and cryoSEM. Using the different capabilities of each method, we gained multifaceted insights into the architecture of peptide fibrils. The results of our comprehensive analysis unveiled compelling insights into the morphology of the RADA-PDGF2 hydrogel. When present in aqueous solutions, the peptide exhibited a propensity to form a network of fibers with dimensions ranging from 200 to 600 nm, as discerned from TEM and AFM images. RADA16-I analogs, similar to the parent peptide, often display a fibrous morphology when examined through TEM and AFM imaging. These analogs exhibit self-assembled structures characterized by elongated nanofibers in TEM pictures and organized nanoscale patterns in AFM images, contributing to their ability to interact with biological systems and materials at the nanoscale level. The length and width of RADA16-I analog nanofibers can vary considerably, with typical lengths ranging from tens of nanometers to several micrometers, and widths falling within the range of 10–20 nm (Rondeau and Bourdon, 2011; Gelain et al., 2020). Notably, the scanning cryoSEM images of a 1% peptide hydrogel showcased a remarkably organized structure, resulting from the self-assembly of the peptide molecules. This arrangement, reminiscent of a honeycomb pattern, became increasingly apparent at lower magnifications. The advanced magnification capabilities of cryoSEM permitted accurate measurements of inter-unit distances within the structure, revealing an approximately 6 μm spacing between successive units. The cryoSEM images obtained are consistent with our previous ones recorded for similar peptide hydrogels (Dzierżyńska et al., 2023). Comparatively, other hydrogels like chitosan, hyaluronic acid, and commercial HEC hydrogels exhibit less organized structures, resembling sponges with irregularly shaped and larger pores (Conovaloff et al., 2011; Kirch et al., 2012; Macdougall et al., 2018). Additionally, non-swellable thiolyne PEG hydrogels also form structures with unregulated architecture. These observations highlight the peptide hydrogel’s unique and well-structured nature because using hydrogels as scaffolds for three-dimensional culture requires adequate pore size. The pores that are visible in the RADA-PDGF2 hydrogel are small enough to allow cells ranging from 10 to 30 μm to settle at their edges and not collapse inside the hydrogel. However, croSEM images of hydrogels that form a structure similar to that formed by RADA16-I peptide hydrogels are known in the literature, e. g., graphene oxide (Serrano-Aroca et al., 2017), or collagen (Pot et al., 2020) but chemical, physical or enzymatic crosslinking is required to obtain a stable structure.

Wound healing is a dynamic process that includes different cell types, e.g., keratinocytes and fibroblasts, and events such as inflammation, proliferation, and migration of these cells (Addis et al., 2020). When developing new biomaterials for wound healing stimulation, evaluating their effect on the processes responsible for the proper course of wound closure is essential. That is why, in this work, we analyzes the effect of RADA-PDGF2 on the proliferation, viability, and migration of human skin cells and collagen synthesis by human fibroblasts. We also evaluated its effect *in vivo* using the mice dorsal skin injury model. Finally, we performed analyses of its cytotoxicity and immunogenicity, which is an important step in assessing the biocompatibility of newly designed materials.

Proliferation is a critical event in wound healing, and wounds cannot heal completely without sufficient levels of cell proliferation. Interestingly, computational analyses showed that if the proliferation rate is not satisfactory, the impact of cell migration on the wound healing process is negligible (Zanca et al., 2022). PDGF-BB is a potent wound healing stimulator and it can increase cell proliferation (White et al., 2021). Similarly, in our previous work, PDGF2 peptide showed pro-proliferative properties towards human fibroblasts and keratinocytes (Deptuła et al., 2020). Other researchers also show that RADA16-I, functionalized with bioactive motifs, can express pro-proliferative properties towards different cell types. For example, RADA16-I with osteogenic growth peptide ALK, osteopontin cell adhesion motif DGR, and RGD binding sequence PGR accelerated the proliferation of mouse osteoblasts (Horii et al., 2007). RADA16-I combined with RADA-PRG increased the proliferation of skin-derived precursors (Wang et al., 2016), and RADA-IKVAV promoted the proliferation and migration of neural stem cells (Zhang et al., 2010). That is why we assumed that RADA-PDGF2 may be able to stimulate the proliferation of human skin cells. The colorimetric analysis results showed that RADA-PDGF2 expresses the most potent pro-proliferative properties towards human 46BR.1N fibroblasts after 72 h of incubation. For human primary dermal fibroblasts isolated from skin samples, the effect was visible only after 72 h of incubation, and it was weaker than for the cell line. In both cases, after 72-h incubation, the pro-proliferative properties of RADA-PDGF2 were stronger than those of the original PDGF2 peptide (Deptuła et al., 2020). 46BR.1N fibroblasts also showed a weaker response to stimulation with RADA16-I. Thus, after 72 h, the effect was more substantial than for the RADA-IM, RADA-GHK, and RADA-KGHK (Dzierżyńska et al., 2023). The opposite situation is visible for HaCaT keratinocytes. Their response for RADA-PDGF2 was weaker than the 46BR.1N fibroblast and was similar in both incubation times. However, PDGF2 showed much stronger pro-proliferative properties, especially after 72 h of incubation (Deptuła et al., 2020). Compared to RADA16-I, the effect was similar, but the stimulation was weaker than RADA16-I, which was functionalized with other bioactive peptides: GHK and KGHK (Dzierżyńska et al., 2023). Interestingly, literature data shows that PDGF-BB stimulates cell division of fibroblasts, but not HaCaT keratinocytes (Park et al., 2014). So, the stronger pro-proliferative action of RADA-PDGF2 on fibroblast may indicate that its mode of action is more similar to the native PDGF-BB protein than those of the PDGF2 peptide. What is more, RADA derivatives can also reduce the proliferation of human cells. For example, Bradshaw et al. (Bradshaw et al., 2014) showed that compared to RADA16-I, RADA-FPG significantly inhibited the proliferation of fibroblasts. However, for RADA-PDGF2, we did not observe any significant reduction in cell proliferation. Additionally, with the use of confocal microscopy and calcein-AM/PI staining, we analyzed the viability of skin cells after 4 days of cell culture of RADA-PDGF2. The results indicate that all of the tested cells remained viable (no dead cells were visible in the field of view), which indicates the safety of tested hydrogels and confirms that it can be potentially used as a scaffold for cell transplantation. The results are comparable to those obtained for RADA-GHK, RADA-KGHK, and RADA-IM, which retained their viability after 3 days of cell culture (Dzierżyńska et al., 2023).

Cell migration is another vital process necessary for a complete wound closure. Already 12 h after injury, keratinocytes start to migrating into the wound bed. During the proliferative phase, fibroblasts also proliferate and migrate, which is essential for rearranging the extracellular matrix (ECM) (Dekoninck and Blanpain, 2019). Although in our previous work, we did not observe significant por-migratory activity of PDGF2 peptide (Deptuła et al., 2020), PDGF-BB is known to stimulate the migration of human fibroblasts (De Donatis et al., 2010). However, for a single cell, both processes can not happen simultaneously. For PDGF-BB, Donatis et al. showed that fibroblasts switch from migrating to proliferating phenotype when they sense an increasing concentration of PDGF (De Donatis et al., 2008). On the other hand, literature data suggests that functionalizations of RADA16-I with bioactive molecules, e.g., collagen type I motif, may result in obtaining scaffolds with pro-migratory properties (Bradshaw et al., 2014). Additionally, Jian et al. (Jian et al., 2022) designed a PDGF-BB supramolecular hydrogel, composed of PDGF epitope VRKIEIVRKK and self-assembling motif derived from *β*-amyloid peptide, which was able to stimulate both proliferation and migration of vascular endothelial cells. For RADA-PDGF2 a significant increase in the migration of HaCaT keratinocytes was observed, whereas for fibroblast the effect was weaker. As RADA-PDGF2 stimulates both, the proliferation and migration of human keratinocytes, its application may result in accelerated epithelialization of the wound and increased wound closure.

Collagen is a main component of the ECM and has an essential role in the regulation of the wound healing process (Mathew-Steiner et al., 2021). The effect of RADA-PDGF2 on collagen synthesis *in vitro* was examined after stimulation of 46BR.1N cells and primary human dermal fibroblasts. Only a small increase of collagen synthesis by human primary fibroblasts was observed, suggesting that RADA-PDGF2 does not stimulate this process significantly in human fibroblasts.

During the development of new biomaterials, it is crucial to analyze not only their physicochemical properties and activity but also their biocompatibility and potential risk to human cells and tissues (Schmalz and Galler, 2017). That is why we decided to evaluate the cytotoxicity of RADA-PDGF2 and its effect on immune cells. Based on our experience and literature data, we know that peptides can be cytotoxic to human cells (Greco et al., 2020). In our previous work, one of the PDGF-BB derivatives, PDGF2-HTT, a heterocyclic form of PDGF2, expressed cytotoxicity to skin cells. However, RADA-PDGF2, similar to PDGF2 and RADA16-I and its other derivatives did not show cytotoxicity to analyzed cells (Deptuła et al., 2020; Dzierżyńska et al., 2023). It indicates that the use of RADA-PDGF2 is safe for human cells and tissues.

Understanding immune response to hydrogels is also crucial during their preclinical analysis. Activation of immune cells may lead to excessive inflammation and consequently, the development of chronic wounds (Fernandez-Yague et al., 2022). Our previous analyses showed that PDGF2 does not cause immune cell activation and does not express the potential to induce allergies (Deptuła et al., 2020). Data obtained for RADA16-I and RADA-PDGF2 indicate its safety in the context of an immune response. However, some significant activation was observed, like an increase of CD69 on CTL cells. Cytotoxic lymphocytes are responsible for eliminating pathogens and can control inflammation and release anti-inflammatory cytokines (Caminero et al., 2023). For RADA-PDGF2, we also observed an increase in CD11^+^CD83^+^ active dendritic cells (Grosche et al., 2020). Expression of CD83 on DCs is crucial for the host’s immune response against bacteria. The observed activation of CTL cells and increase in CD83^+^ DCs may be helpful in the treatment of infected wounds.

Both PDGF-BB and PDGF2 applied in p407 accelerated wound healing (Deptuła et al., 2020). RADA16-I was also reported to stimulate wound healing, e.g., deep second-degree burns in rats (Meng et al., 2009) or in a sheep model of endoscopic nasal surgery (Lee and Ananda, 2023). To analyze the effect of RADA-PDGF2 on wound healing, we evaluated its effect in mice dorsal skin injury model. The analysis showed that compared to RADA16-I (control), RADA-PDGF2 accelerated both wound closure and epithelialization. In both cases, differences were significant from the day of the seventh after injury. Interestingly, the mean percentage of wound closure for RADA-PDGF2 treated mice on day 7 was bigger than for previously analyzed RADA-IM, RADA-GHK, and RADA-KGHK (Dzierżyńska et al., 2023). Faster wound closure and restoration of the epidermis help avoid wound healing complications like bacterial infections, which in turn may lead to the development of chronic wounds (Azmat and Council, 2023). Finally, RADA16-I, known as PuraDermTM is registered in the US as wound dressing (Sankar et al., 2021), so the results obtained for RADA-PDGF2 showing it accelerates wound healing compared to this hydrogel, indicate its clinical potential. However, further research is necessary.

In conclusion, the novel self-assembling RADA-PDGF2 hydrogel demonstrates promising potential for advancing wound healing therapies. As a self-assembling gelling peptide, it does not require an additional carrier, which is an essential advantage. This scaffold exhibits excellent stability in human plasma and extreme temperature resilience. Notably, it undergoes precise cleavage by neutrophil elastase, releasing the bioactive PDGF2 motif. Importantly, it proves biocompatible, as it does not induce cytotoxicity in human cells and avoids excessive immune cell activation, ensuring immunological safety. Furthermore, RADA-PDGF2 fosters the proliferation and migration of skin cells and encourages collagen synthesis in fibroblasts, making it a promising candidate for cell transplantation scaffolding. The observed activation of CTLs and DCs suggests its potential utility in infected wounds, albeit further comprehensive research is warranted. Ultimately, RADA-PDGF2 accelerates wound closure and epithelialization, positioning it as a compelling candidate for enhancing wound healing applications. However, further research is needed, especially regarding the issue of neovascularization after application of RADA-PDGF2.

## Data Availability

The data presented in the study is deposited in the Zenodo respiratory https://doi.org/10.5281/zenodo.10159122.
