# Supplementary material for: Development and evaluation of RADA-PDGF2 self-assembling peptide hydrogel for enhanced skin wound healing
Source: Front Pharmacol. 2023 Nov 28;14:1293647. doi: 10.3389/fphar.2023.1293647 (PMC10716921; doi:10.3389/fphar.2023.1293647)
Supplement: Supplementary file 1 [file Table2.DOCX]

***Supplementary Material***

**Development and evaluation of RADA-PDGF2 self-assembling peptide hydrogel for enhanced skin wound healing**

**Deptuła M^1*^**^†^**, Sawicka J^2*^**^†^**, Sass P^3^, Sosnowski P^3^, Karpowicz P^4^, Zawrzykraj M^5^, Wardowska A^1,6^, Tymińska A^1^, Dzierżyńska M^2^, Pietralik-Molińska Z^7^, Peplińska B^8^, Zieliński J^9^, Kondej K^10^, Kozak M^7^, Sachadyn P^3^, Rodziewicz-Motowidło S^2^, Pikuła M^1^**

*- Corresponding Authors

†- equally contributed

^1^ Laboratory of Tissue Engineering and Regenerative Medicine, Division of Embryology, Medical University of Gdańsk, Gdańsk, Poland

^2^ Department of Biomedical Chemistry, Faculty of Chemistry, University of Gdańsk, Gdańsk, Poland

^3^ Laboratory for Regenerative Biotechnology, Faculty of Chemistry, Gdańsk University of Technology, Gdańsk, Poland

^4^ Department of Organic Chemistry, Faculty of Chemistry, University of Gdańsk, Gdańsk, Poland

**^5^** Division of Clinical Anatomy, Medical University of Gdańsk, Gdańsk, Poland

^6^ Department of Physiopathology, Faculty of Medicine, Medical University of Gdańsk, Poland

^7^Department of Macromolecular Physics, Faculty of Physics, Adam Mickiewicz University, Poznań, Poland

^8^ NanoBioMedical Centre, Adam Mickiewicz University, Poznań, Poland

^9^ Department of Surgical Oncology, Medical University of Gdańsk, Gdańsk, Poland

^10^ Department of Plastic Surgery, Medical University of Gdańsk, Gdańsk, Poland

*** Correspondence:**Deptuła Milena, PhD Eng.
[milena.deptula@gumed.edu.pl](mailto:milena.deptula@gumed.edu.pl)

Sawicka Justyna, PhD

[justyna.sawicka@ug.edu.pl](mailto:justyna.sawicka@ug.edu.pl)

**Keywords: hydrogels, wound healing, skin, RADA16-I, PDGF, peptides, croSEM**

## Supplementary Figures


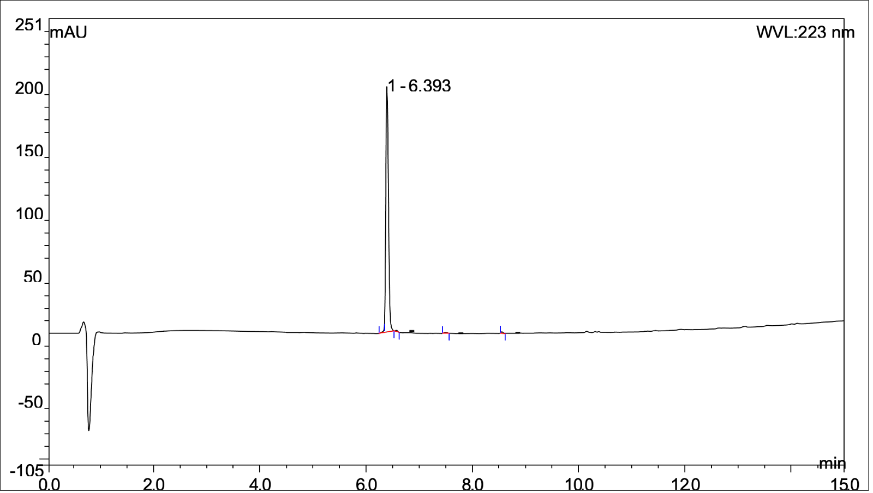


Figure S1. The UHPLC chromatogram presented in Figure 1 showcases the analytical separation of pure RADA-PDGF2 peptide, providing insight into its molecular composition and purity.


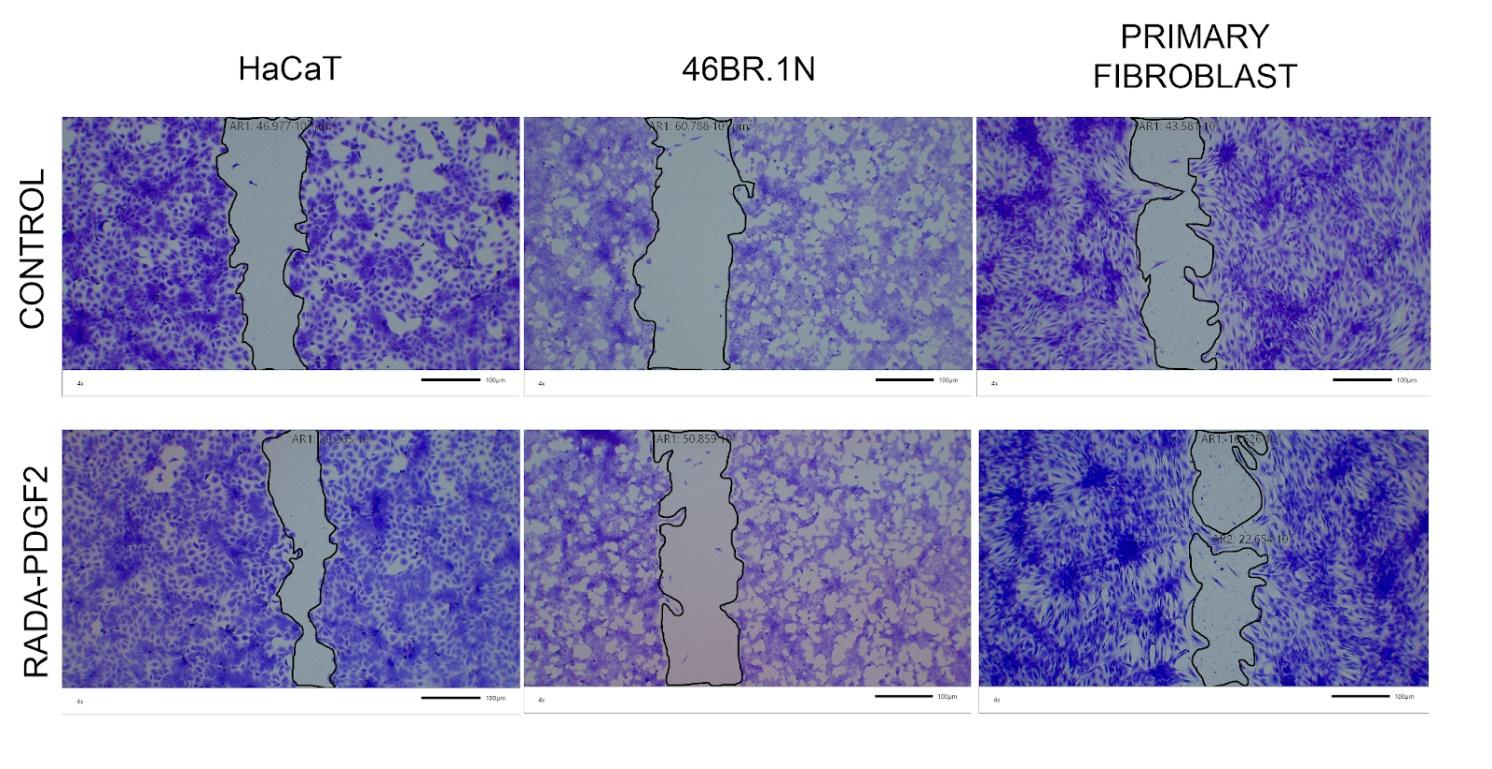


Figure S2. Representative images of cell migration analysis after 24h incubation with RADA-PDGF2.

**2.2 Supplementary tables**

Table S1. Most abundant signals in mass spectrometry correspond to identified peptide fragments after elastase digestion.

| **The sequence of identified fragments** | **Theoretical monoisotopic mass [Da]** | **Experimental monoisotopic mass [Da]** |
| --- | --- | --- |
| G^24^GGRLIDRTNA^34^ | 1128.40 | 1128.60 |
| R^1^ADARADARADA-RADAGGGAAPV^23^ | 2222.09 | 2221.70 |
| G^24^GGRLIDRTNANFL^37^ | 1501.81 | 1501.54 |
